# Supplementary material for: Understanding the Functional Properties of Lipid Heterogeneity in Pulmonary Surfactant Monolayers at the Atomistic Level
Source: Front Cell Dev Biol. 2020 Nov 16;8:581016. doi: 10.3389/fcell.2020.581016 (PMC7701215; doi:10.3389/fcell.2020.581016)
Supplement: Supplementary file 1 [file Data_Sheet_1.pdf]

Supporting Information for: Understanding the Functional Properties of Lipid Heterogeneity in Pulmonary Surfactant Monolayers at the Atomistic Level

Juho Liekkinen,[†,‡] Berta de Santos Moreno,[¶,‡] Riku O. Paananen,[§] Ilpo Vattulainen,[*,†,‖,⊥] Luca Monticelli,[*,#] Jorge Bernardino de la Serna,[*,¶] and Matti Javanainen[*,@,†,△]

†*Department of Physics, University of Helsinki, FI-00014 Helsinki, Finland*

‡*These authors contributed equally*

¶*National Heart & Lung Institute, Faculty of Medicine, Imperial College London, SW7 2AZ, London, UK*

§*Helsinki Eye Lab, Ophthalmology, University of Helsinki and Helsinki University Hospital, FI-00014 Helsinki, Finland*

‖*Computational Physics Laboratory, Tampere University, FI-33014, Tampere, Finland*

⊥*MEMPHYS – Centre for Biomembrane Physics*

#*Molecular Microbiology and Structural Biochemistry (MMSB), UMR 5086 CNRS & University of Lyon, Lyon, France*

@*Laboratory of Physics, Tampere University, FI-33014, Tampere, Finland*

△*Institute of Organic Chemistry and Biochemistry of the Czech Academy of Sciences, CZ-16100 Prague 6, Czech Republic*

E-mail: ilpo.vattulainen@helsinki.fi; luca.monticelli@inserm.fr; j.bernardino-de-la-serna@imperial.ac.uk; matti.javanainen@gmail.com

Simulation Details

Details on Simulation Models and Methods

Four component lipid monolayers mimicking the native lipid composition of the pulmonary surfactant were simulated with different values for the average area per lipid (APL). A system containing two monolayers with a quaternary mixture of 60 mol-% dipalmitoylphosphatidylcholine (DPPC), 20 mol-% 1-palmitoyl-2-oleoylphosphatidylcholine (POPC), 10 mol-% 1-palmitoyl-2-oleoylphosphatidylglycerol (POPG), and 10 mol-% cholesterol, with a total of 338 lipids (169 per monolayer), a total of 27 040 water molecules (80 per lipid), and an ion concentration of 0.15 M was obtained from the CHARMM-GUI monolayer builder. The initial area per lipid of the system was calculated by CHARMM-GUI from its suggested lipid area values (equal to approximately 62 $Å^2$). The suggested equilibration scheme for Gromacs was used with the included constraints and simulation parameters, including minimization steps, constrained simulations in the NVT ensemble, and equilibration simulations in the NPT ensemble. During the last steps of the suggested equilibration scheme, the monolayer area per lipid (APL) settled to an initial configuration of approximately 50 $Å^2$. At this point, the monolayers were separated by a slab of water with air (vacuum) on both sides of the system. The box size in the z direction was set to 22.00 nm to avoid unscreened electrostatic interactions through the vacuum. After the initial equilibration, the original TIP3 water molecules were changed to the 4-point OPC[1] molecules, followed by a minimization, constrained equilibration, and an unconstrained 50 ns simulation in the NVT ensemble at 310 K, using the suggested simulation parameters for the CHARMM36+OPC combination.[2]

Next, the monolayer structure was either expanded, or compressed during a 10 ns simulation to an average APL value of 100 $Å^2$, or 40 $Å^2$, respectively, using the `MOVINGRESTRAINT` and `CELL` keywords in the PLUMED 2.2 package.[3] From the expansion simulation, frames corresponding to APLs of 52.5, 55, 57.5, 60, 62.5, 65, 67.5, 70, 75, 80, 85, 90, 95, and 100 $Å^2$ were extracted. From the compression simulations, frames corresponding to APLs of 47.5,

and 45 were extracted. Independent repetitions at selected APLs of 55, 65, and 75 Å^2 were also performed from similarly built initial configurations.

These quaternary lipid monolayers were simulated at both 310 K and 298 K for 1.0 µs. The suggested parameter set for CHARMM36 force field combined with the OPC4 water model were used in all monolayer simulations. The simulations were performed in the NVT ensemble and the dispersion correction[4] was applied to both energy and pressure.

We also repeated selected simulations using larger monolayer models. The initial structures for the large quaternary lipid monolayers were taken as the final configurations of selected smaller monolayer simulations described above. The structures were multiplied using the `g_genconf` tool, to generate a system with a total of 1352 lipids (676 per monolayer) and a total of 108160 water molecules, and a system with a total of 3042 lipids (1521 per monolayer) and a total of 243360 water molecules. All systems had an ion concentration of 0.15 M. The simulation parameters were identical to the ones used with the small monolayers. The 1352 lipid systems with APLs of 50.0 and 55.0 Å^2 at 298 K and 310 K, respectively, were simulated for 1 µs both. The 3042 lipid system with APL of 55.0 Å^2 at 310 K was simulated for 500 ns.

Calculation of γ_0

The pure air–water interface was simulated using the four-point OPC[1] (OPC4) water model to evaluate the interfacial surface tension values (γ_0) used in the calculation of the surface pressure of the monolayers. The systems contained 27040 water molecules for the smaller, and 108160 water molecules for the larger systems, with box dimensions of $9.19^2 \times 22.00$ nm^3, and $19.28^2 \times 22.00$ nm^3, respectively, corresponding to the average sizes of the monolayer simulations described above. The OPC4 water model with the suggested simulation parameters for the CHARMM36 force field with GROMACS was used (see Ref. 2 for details). The systems containing 27040 water molecules were simulated at 298 K and 310 K for 30 ns, and the systems with 108160 water molecules were simulated at 298 K and 310 K for 10 ns.

The simulations were performed in the NVT ensemble and the dispersion correction[4] was applied to both energy and pressure. The first 10 ns of the simulations were omitted from the analysis of the smaller water systems and the first 1 ns were omitted from the analysis of the larger water systems. All simulations were run with GROMACS 5.1.x.[5] The surface tension of water (γ_0) was extracted using the `g_energy` tool.

Supplementary Experimental Results

Pressure–Area Isotherms

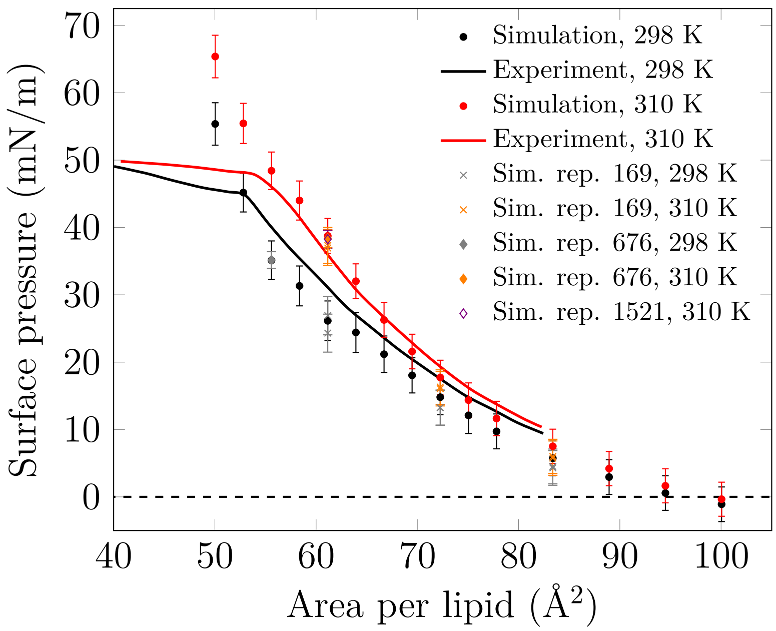

Figure S1: Surface pressure–Area isotherms at 298 K and 310 K. Due to the periodic boundary conditions and the finite sizes of the systems, the monolayers cannot collapse in the simulated time scale. Therefore, the simulations overestimate the surface pressures of the quaternary monolayers with areas below 53 and 56 Å^2 at 298 K and 310 K, respectively, where the monolayers are mostly in the L_c phase and in a metastable state. Replicas were performed for certain APL values to check the consistency of the calculated surface pressure values. Moreover, additional simulations with larger monolayers (676 or 1521 lipids per monolayer) were performed to evaluate the finite-size effects on the calculated surface pressure values.

AFM Imaging

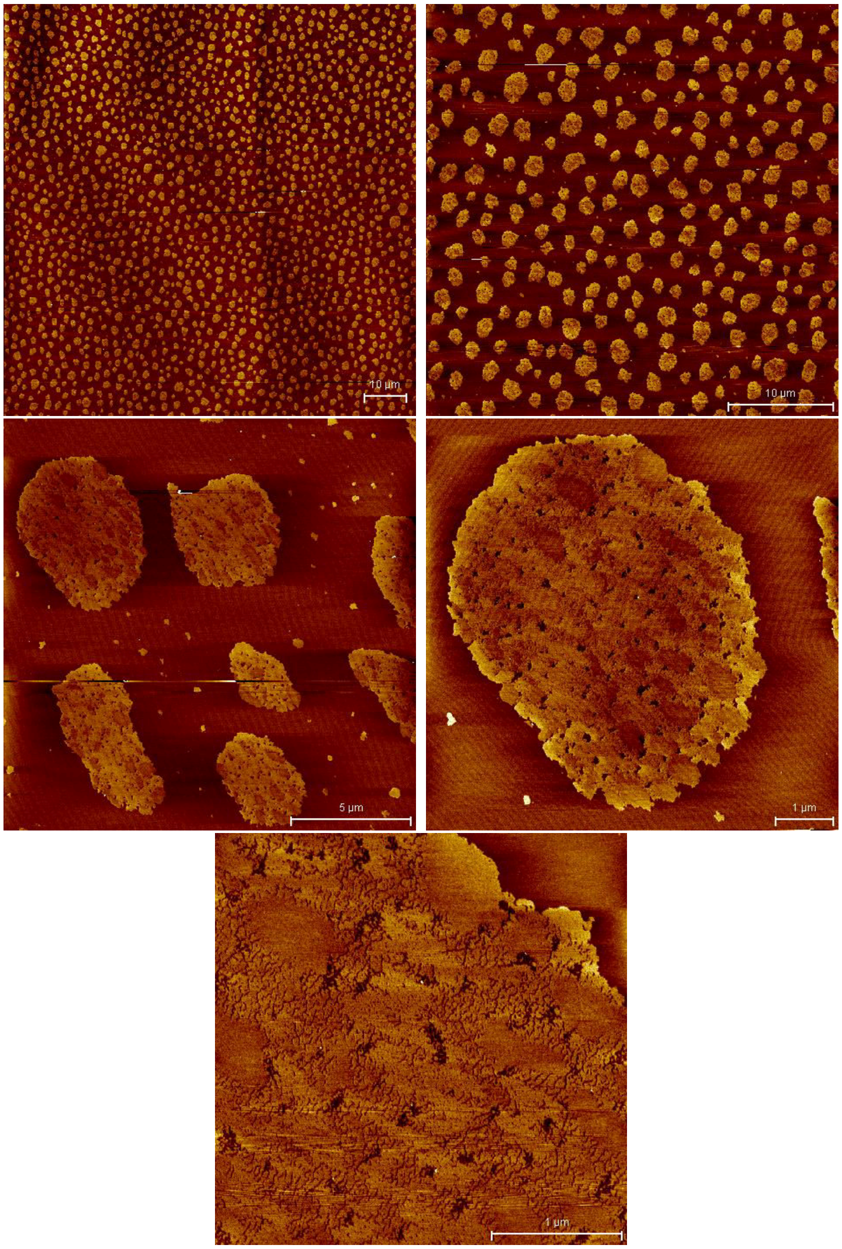

Figure S2: AFM images at 25 mN/ at 298 K.

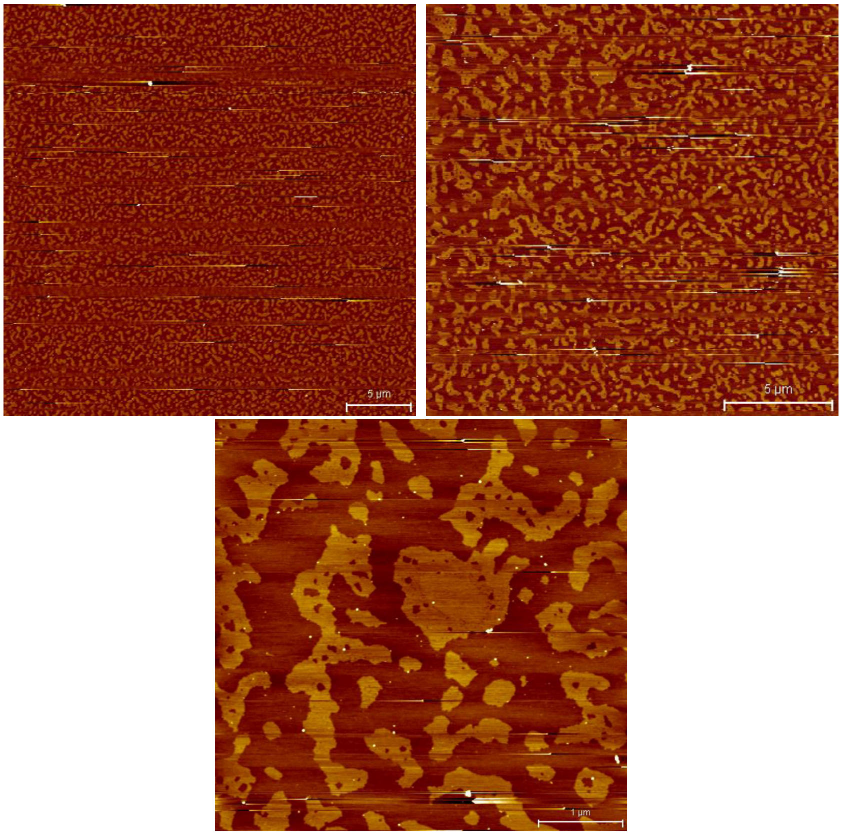

Figure S3: AFM images at 35 mN/m at 298 K.

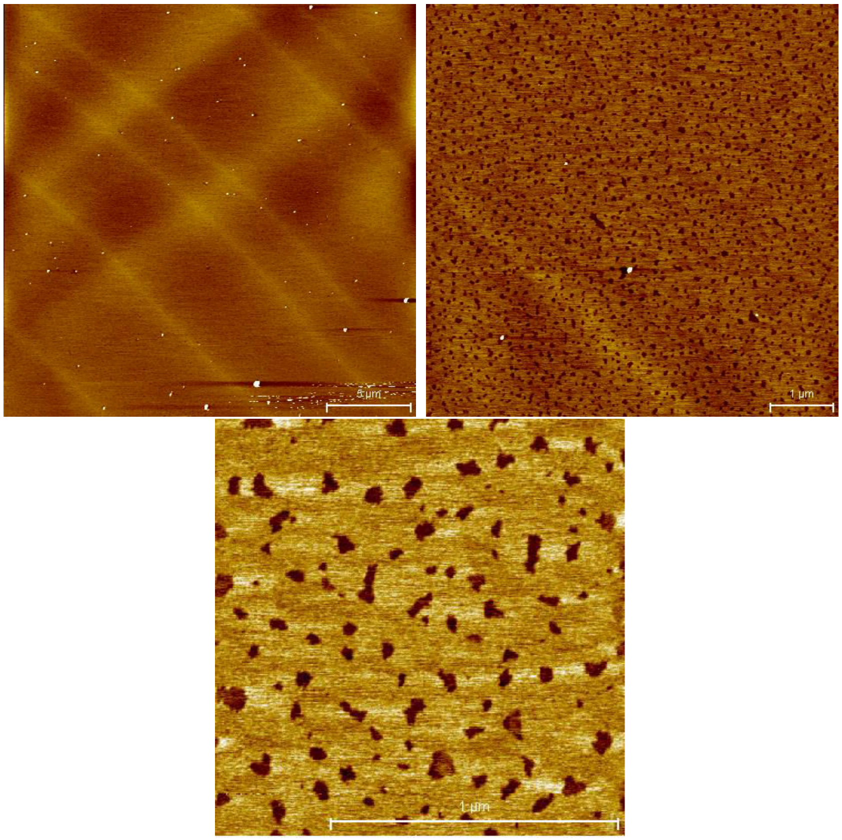

Figure S4: AFM images at 45 mN/m at 298 K.

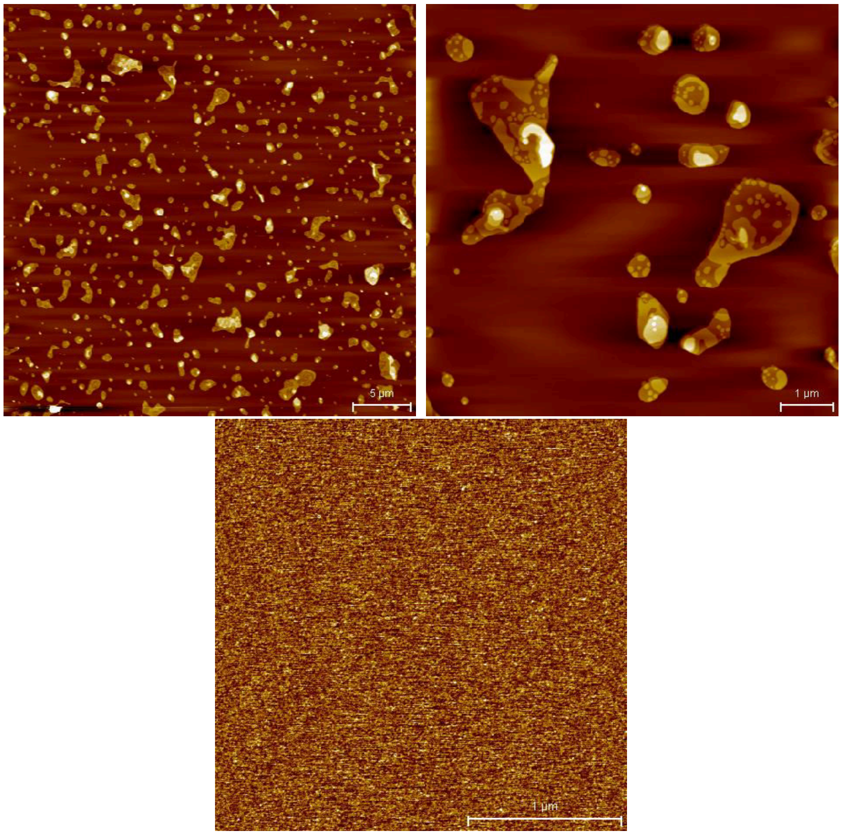

Figure S5: AFM images at 55 mN/m at 298 K.

Height Profile Studies

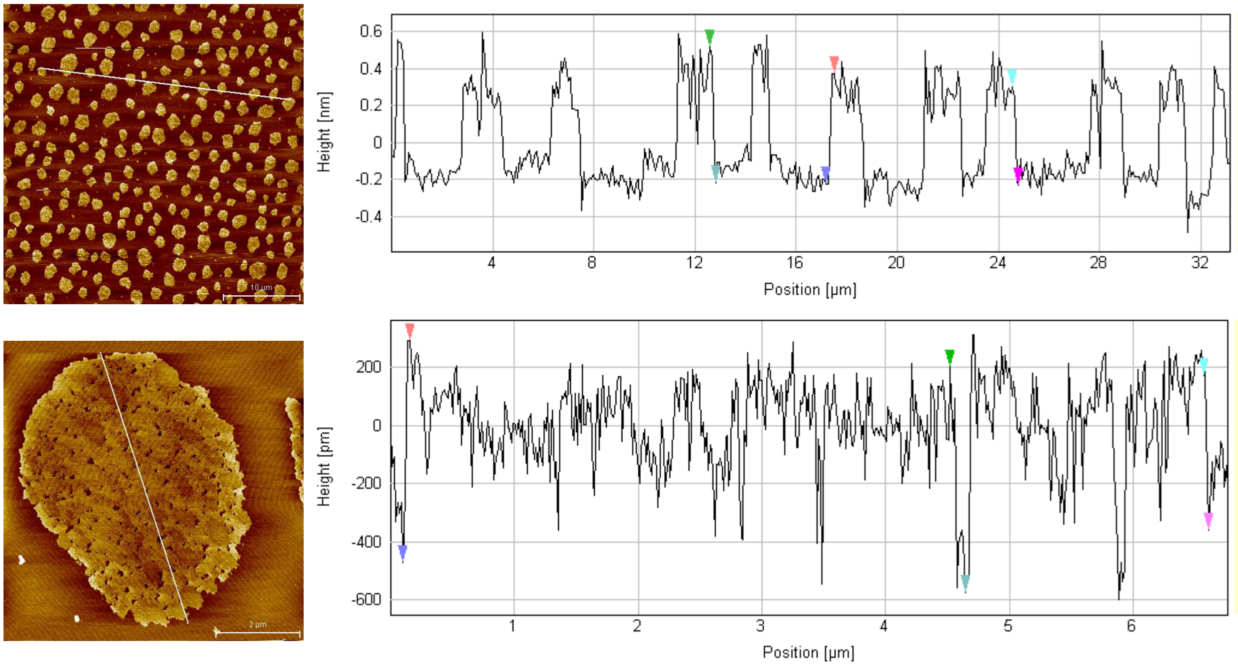

Figure S6: Heigth profile study at 25 mN/m at 298 K.

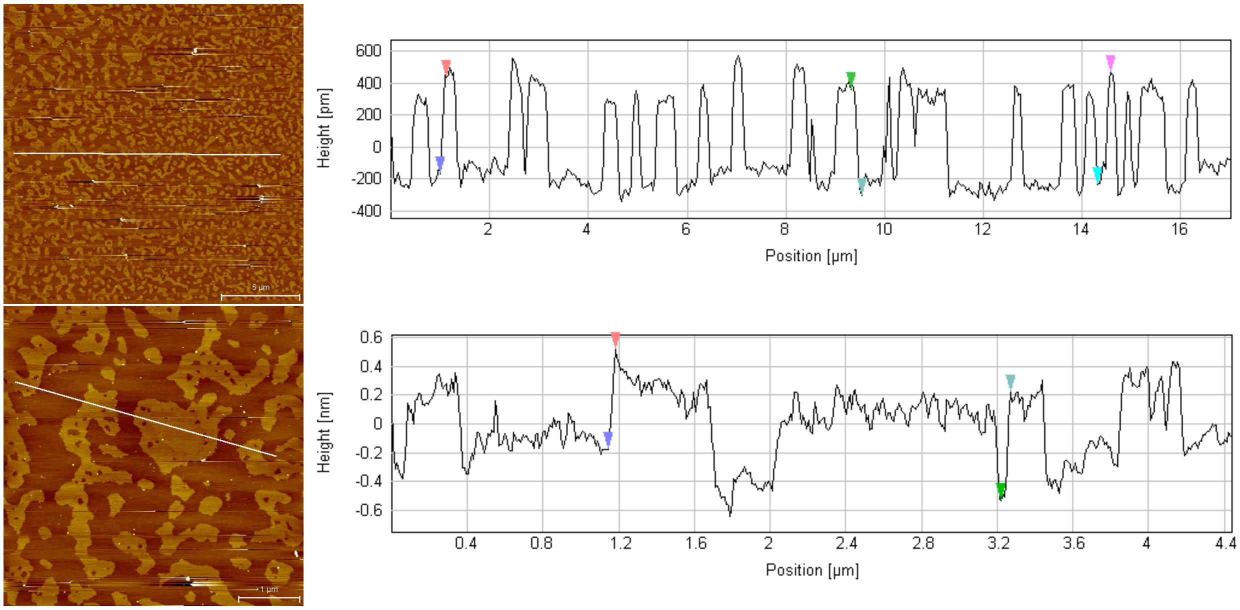

Figure S7: Heigth profile study at 35 mN/m at 298 K.

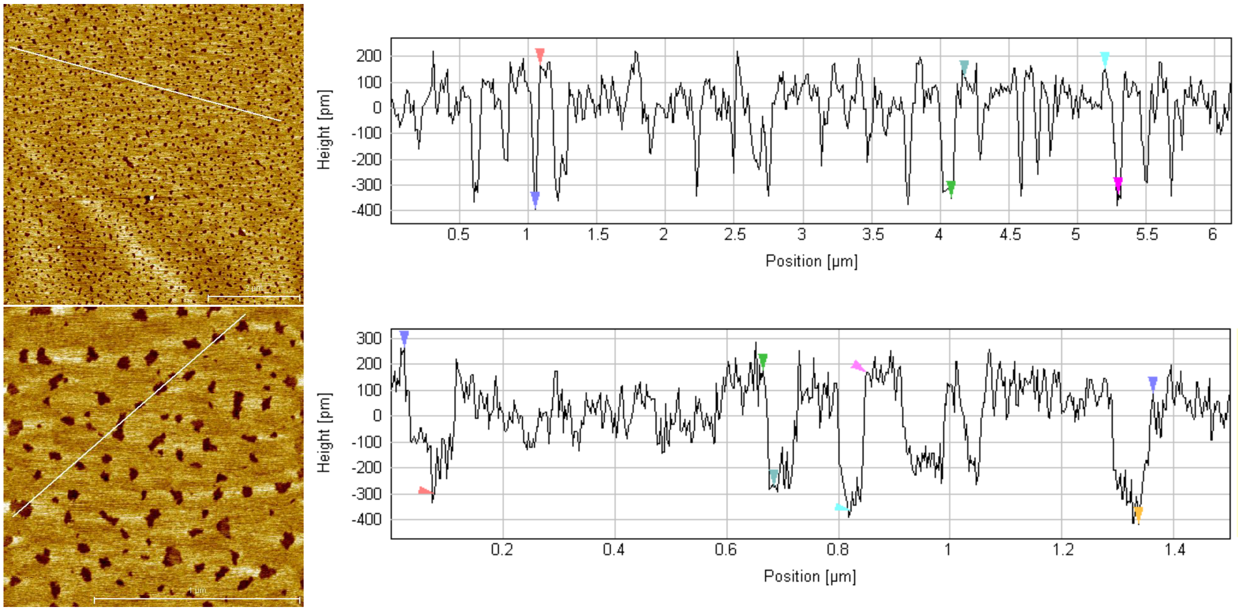

Figure S8: Heigth profile study at 45 mN/m at 298 K.

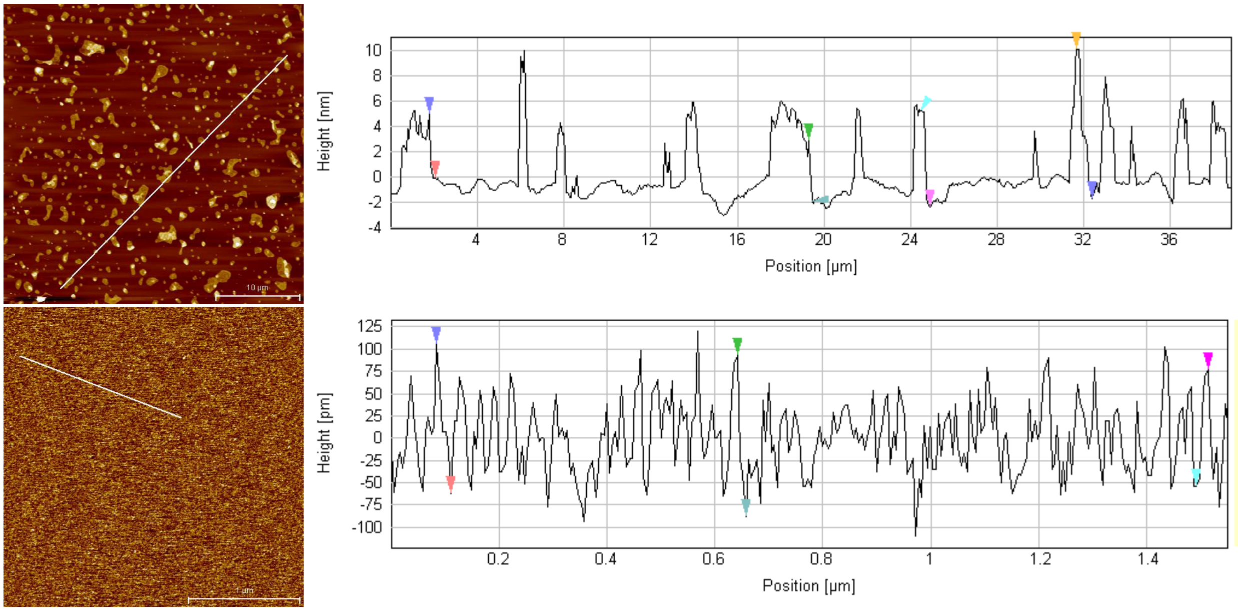

Figure S9: Heigth profile study at 55 mN/m at 298 K.

Supplementary Simulation Results

Snapshots of the Lipid Monolayer Simulations

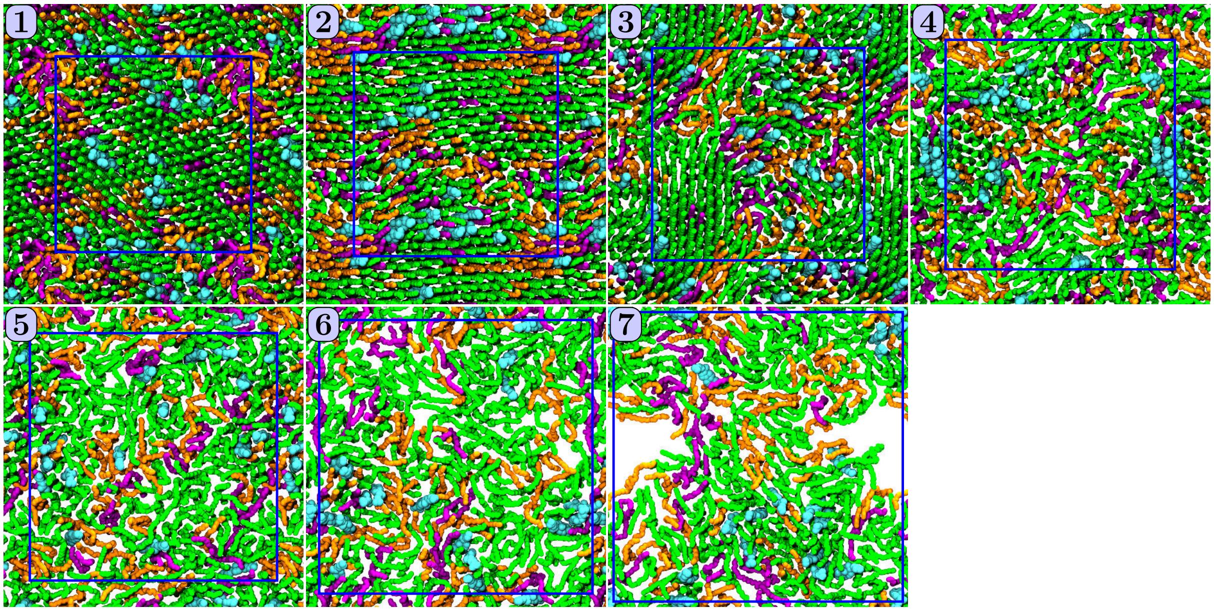

Figure S10: Snapshots of the lipid monolayers at 298 K. Top row, from left to right: 45 Å^2, 50 Å^2, 55 Å^2, and 65 Å^2. Bottom row: 75 Å^2, 90 Å^2, and 100 Å^2. Blue lines highlight the simulation cell.

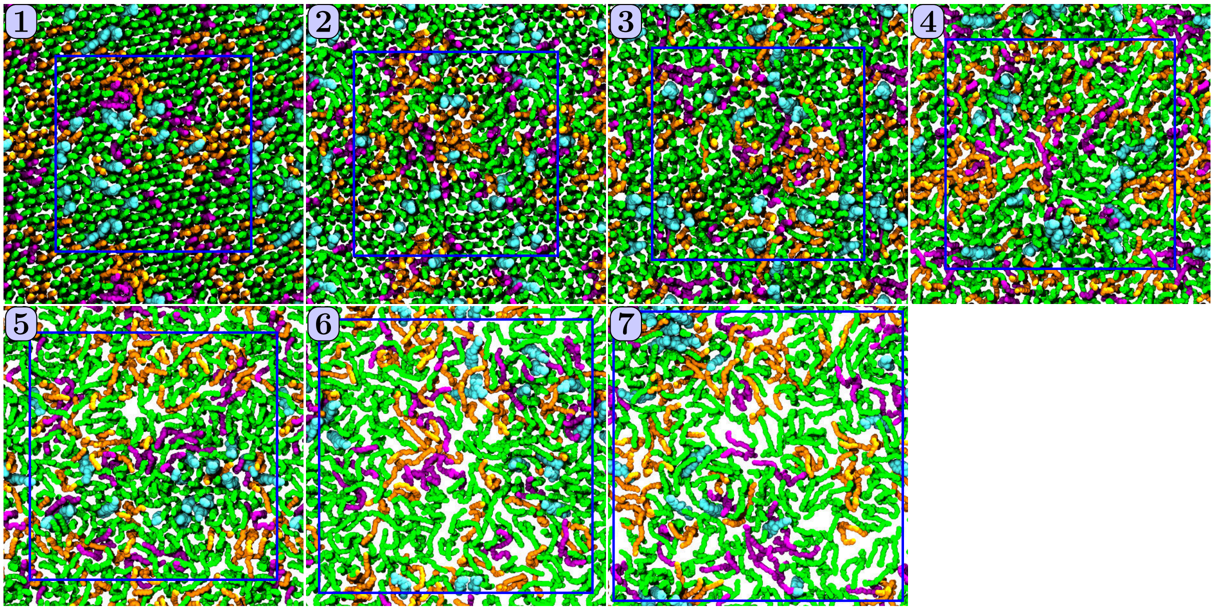

Figure S11: Snapshots of the lipid monolayers at 310 K. Top row, from left to right: 45 Å^2, 50 Å^2, 55 Å^2, and 65 Å^2. Bottom row: 75 Å^2, 90 Å^2, and 100 Å^2. Blue lines highlight the simulation cell.

Tilt Angle Distributions

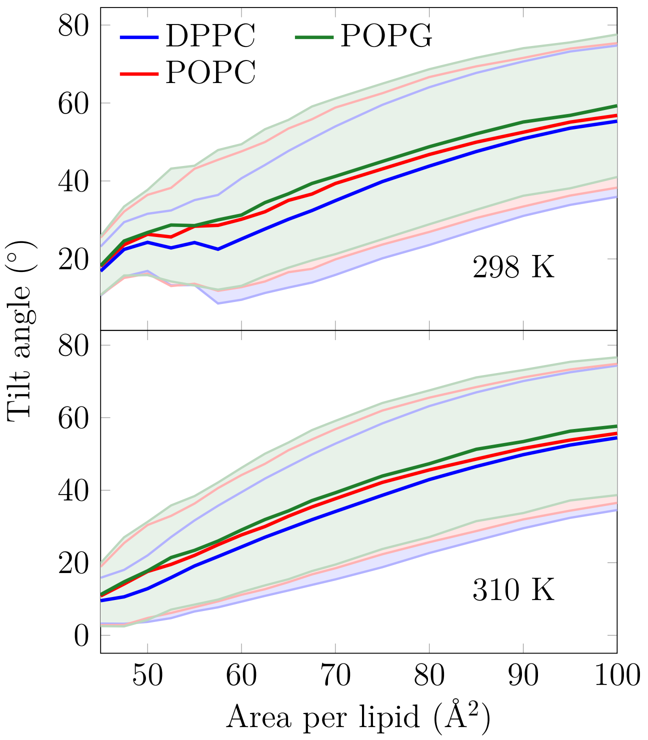

Figure S12: Tilt angle distributions of lipid chains. The shaded areas, bordered by dim lines, show the standard deviation.

Cholesterol clustering

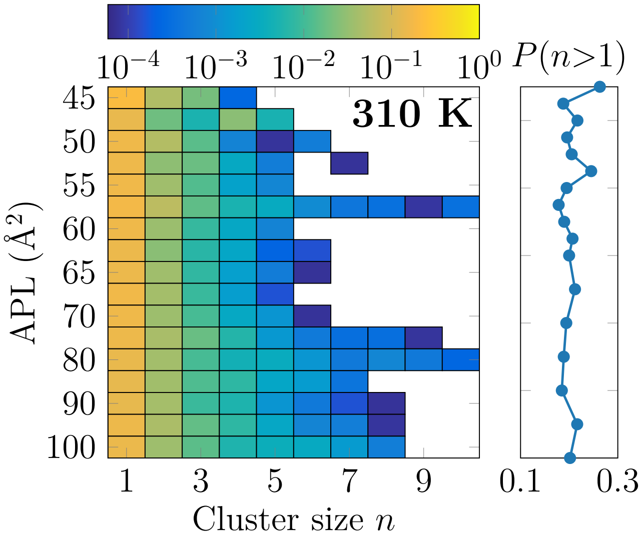

Figure S13: Left: 2-dimensional probability distributions of cholesterol molecules residing in a cluster with at least one other cholesterol at all simulated APLs at 310 K. Note that the color bar is in logarithmic scale. Right: The fraction of cholesterol molecules in clusters with size larger than one.

Effect of DBSCAN parameters

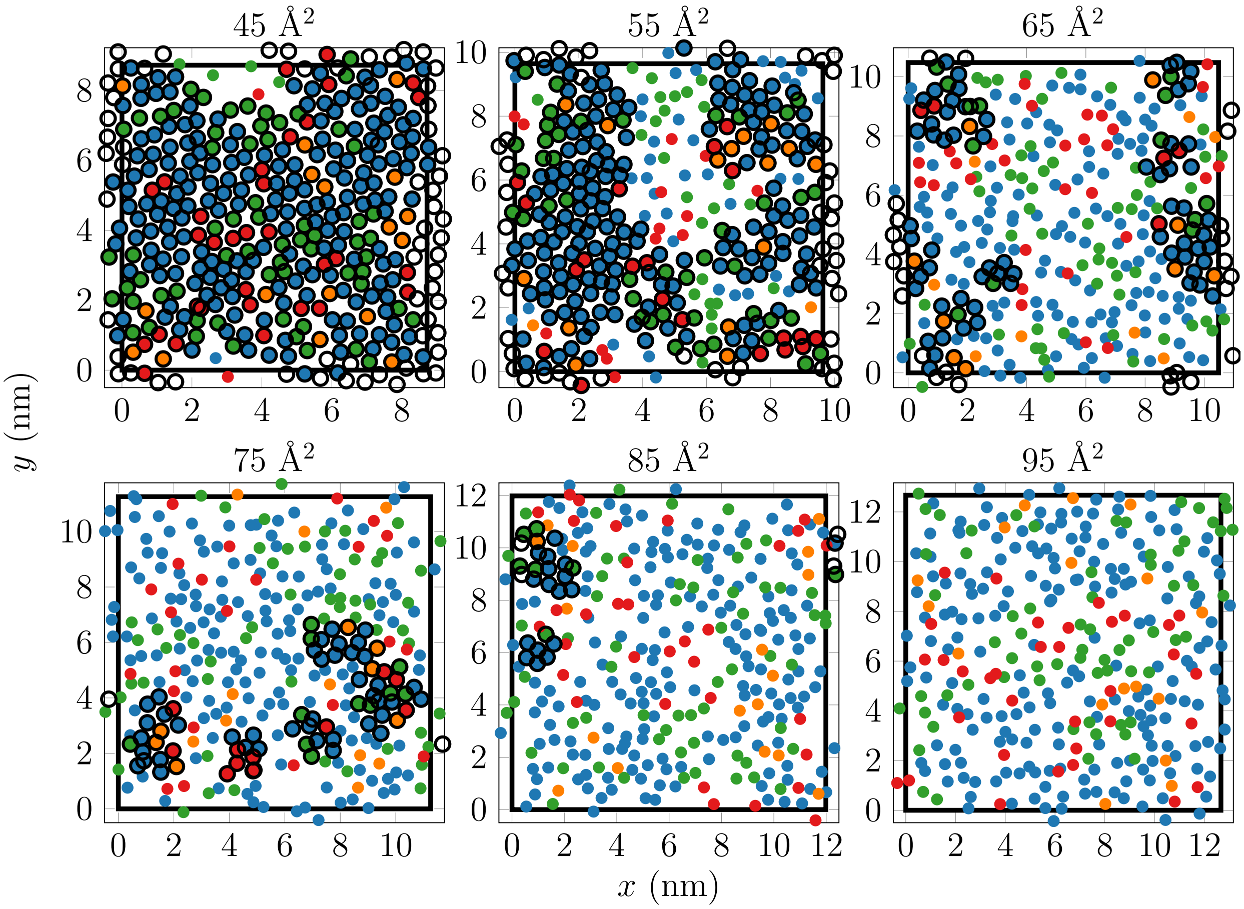

Figure S14: Characteristic snapshots of the clusters detected by the DBSCAN algorithm. The clustering is performed for the simulations performed at 310 K and at different APLs, indicated on top of the panels. Each DPPC chain is represented in blue, each POPC chain in green, each POPG chain in red, and each CHOL molecule in orange. The lipids that are surrounded by a black circle are considered to be part of an L_c-like domain. At the smallest area, almost all lipids are part of the L_c-like clusters, whereas at the largest area, sometimes no lipids are found in these clusters, yet the DBSCAN algorithm can still detect small clusters due to density fluctuations. The simulation box is drawn in black. The periodic images of the lipids are included in DBSCAN. If a periodic image is assigned to an L_c-like cluster but a lipid was already considered on the other side of the simulation box, a black empty circle is drawn.

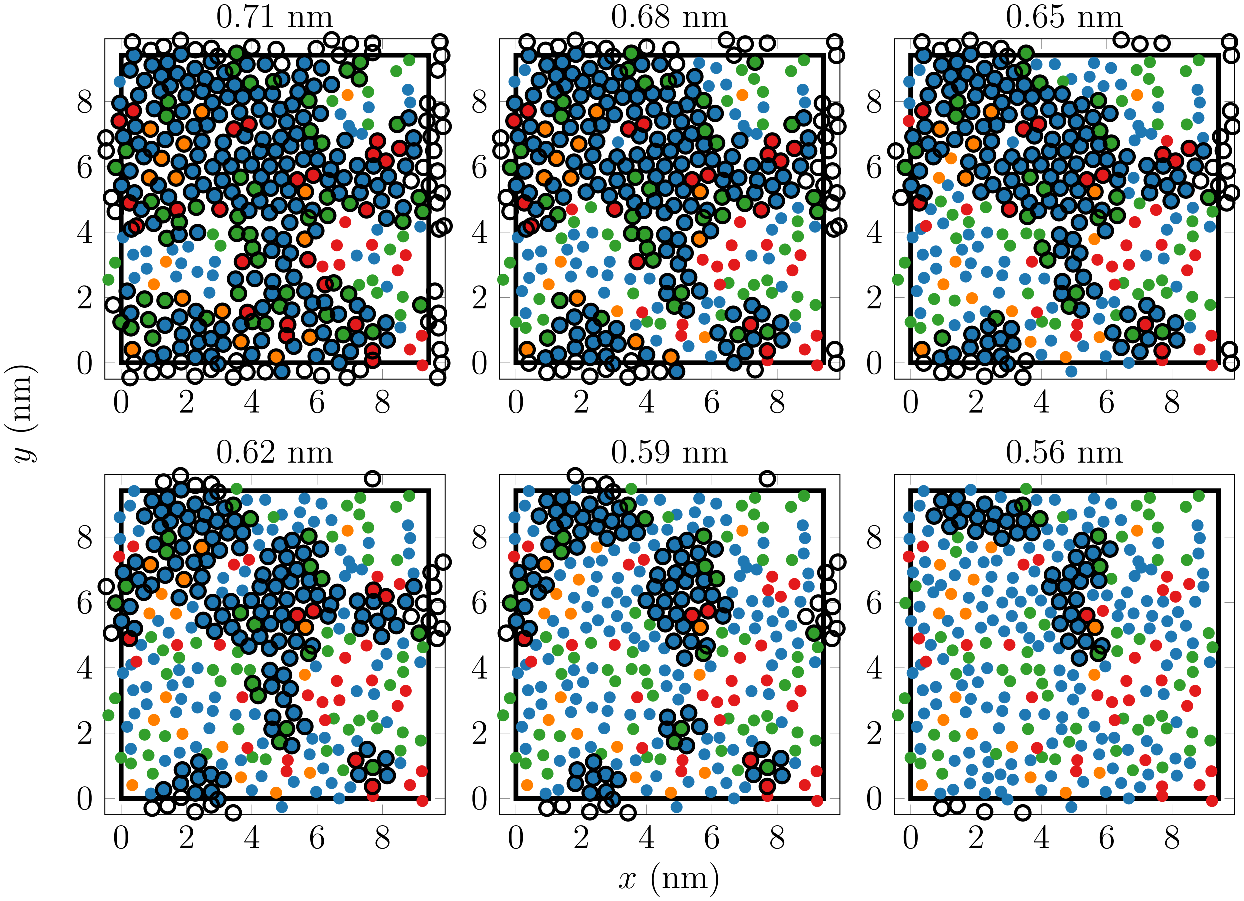

Figure S15: Effect of DBSCAN cutoff on the detected domain structure. The analyzed simulation had APL equal to 52.5 Å^2 and was simulated at 310 K. The used cutoff is indicated on top of the panels. Coloring is as in Fig. S14. Unsurprisingly, the DBSCAN parameters significantly affect the output of the clustering. Our choice of cutoff (0.71 nm) was based on the first minimum in the radial distribution function of the clustered atoms. With this choice, we obtained meaningful results: the systems with the smallest APLs resembled mainly but not fully the L_c-like packing, wheras the systems with the highest APLs had no significant L_c-like clusters (see Fig. S14).

Monolayer Isothermal Compressibility

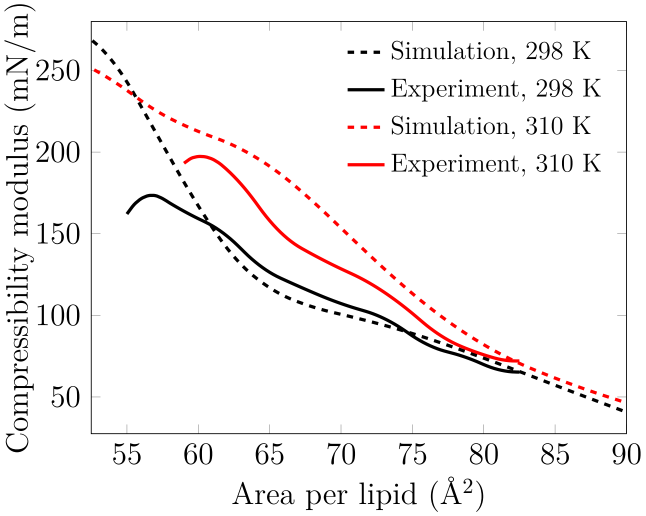

Figure S16: The inverse of the isothermal compressibility ($C_s{}^{-1}$) of the monolayers with respect to the area per lipid. The values are calculated from the surface pressure–area isotherms as $C_s{}^{-1} = -A\frac{d\pi}{dA}$, where A is the average area per molecule and π is the surface pressure. Smoothing parameters of 0.01 and 0.25 were used for the smoothing spline fit in Matlab for the simulated and measured isotherms, respectively. For the measured systems, data are shown for the part of the isotherm where the second derivative of the fit is positive *i.e.* where the slope of the isotherm is increasing towards lower area per lipid.

Monolayer Thickness

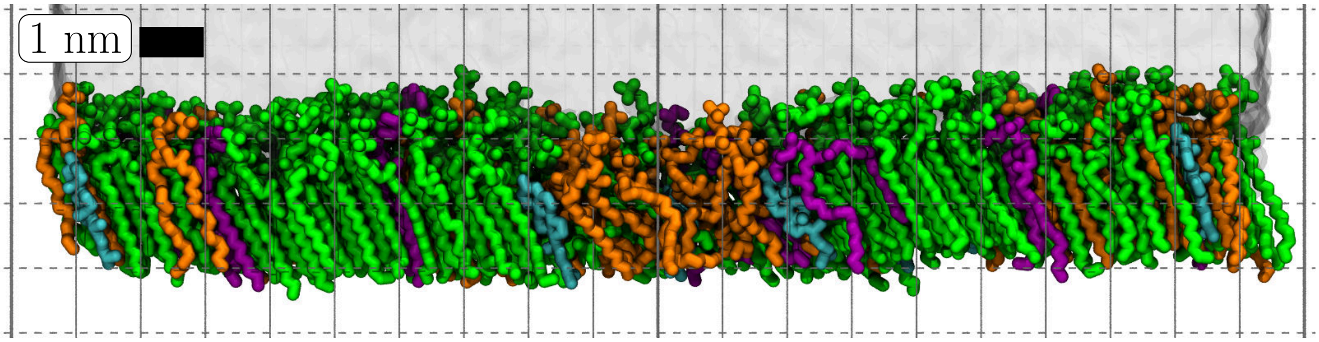

Figure S17: The simulations show a clear difference in the thickness of the L_C-like and L_E phase regions in the monolayers. In this case, a larger 676-lipid-per-monolayer simulation system at 298 K and APL 50 Å^2 shows a disordered and thinner L_E region in the middle, surrounded by the thicker L_C-like regions. DPPC, POPC, POPG, and cholesterol are colored in green, orange, purple, and cyan, respectively. The gray area on top represents the water bulk in between the two monolayers. The black scale bar is equal to 1 nm.

Availability of Simulation Data

Simulation data of the monolayers together with the related files are available online: Part 1/4 containing all small monolayers with APLs 45–55 Å^2 in Ref. 6, Part 2/4 containing all small monolayers with APLs 57.5–67.5 Å^2 in Ref. 7, Part 3/4 containing all small monolayers with APLs 70–90 Å^2 in Ref. 8, and Part 4/4 containing all small monolayers with APL 100 Å^2, and all larger monolayers in Ref. 9.

References

(1) Izadi, S.; Anandakrishnan, R.; Onufriev, A. V. *J. Phys. Chem. Lett.* **2014**, *5*, 3863–3871.

(2) Javanainen, M.; Lamberg, A.; Cwiklik, L.; Vattulainen, I.; Ollila, O. S. *Langmuir* **2017**, *34*, 2565–2572.

(3) Tribello, G. A.; Bonomi, M.; Branduardi, D.; Camilloni, C.; Bussi, G. *Comput. Phys. Commun.* **2014**, *185*, 604–613.

(4) Shirts, M. R.; Mobley, D. L.; Chodera, J. D.; Pande, V. S. *J. Phys. Chem. B* **2007**, *111*, 13052–13063.

(5) Abraham, M.; Murtola, T.; Schulz, R.; Páll, S.; Smith, J.; Hess, B.; Lindah, E. *SoftwareX* **2015**, *1-2*, 19–25.

(6) Liekkinen, J.; Javanainen, M. DPPC/POPC/POPG/CHL1 Monolayer Simulations With Charmm36+OPC (Part 1/4). 2020; `http://10.5281/zenodo.3898344`.

(7) Liekkinen, J.; Javanainen, M. DPPC/POPC/POPG/CHL1 Monolayer Simulations With Charmm36+OPC (Part 2/4). 2020; `http://10.5281/zenodo.3899875`.

(8) Liekkinen, J.; Javanainen, M. DPPC/POPC/POPG/CHL1 Monolayer Simulations With Charmm36+OPC (Part 3/4). 2020; `http://10.5281/zenodo.3899535`.

(9) Liekkinen, J.; Javanainen, M. DPPC/POPC/POPG/CHL1 Monolayer Simulations With Charmm36+OPC (Part 4/4). 2020; http://10.5281/zenodo.4034250.
